# Evaluation of Disease Suppressiveness of Soils in Croplands by Co-Cultivation of Pathogenic *Fusarium oxysporum* and Indigenous Soil Microorganisms

**DOI:** 10.1264/jsme2.ME21063

**Published:** 2022-10-01

**Authors:** Masahiro Mitsuboshi, Yuuzou Kioka, Katsunori Noguchi, Susumu Asakawa

**Affiliations:** 1 Tsukuba Research Institute, Katakura & Co-op Agri Corporation, 5–5511 Namiki, Tsuchiura, Ibaraki 300–0061, Japan; 2 Katakura & Co-op Agri Corporation, 1–8–10 Kudankita, Chiyoda, Tokyo 102–0073, Japan; 3 Graduate School of Bioagricultural Sciences, Nagoya University, 1 Furo-cho, Chikusa, Nagoya, Aichi 464–8601, Japan

**Keywords:** suppression of soil-borne disease, biological diagnosis, disease incidence, spinach wilt

## Abstract

An evaluation of suppressiveness against soil-borne diseases is important for their control. We herein assessed disease suppression against *F. oxysporum* f. sp. *spinaciae* using the *Fusarium* co-cultivation method in 75 soils collected from croplands around the country. The suppressive effects of soil microbes against *F. oxysporum* growth were examined by simultaneously culturing soil suspensions and *F. oxysporum* f. sp. *spinaciae* on a culture medium. The growth degree of *F. oxysporum* on the medium varied among the 75 soils tested, and 14 soils showing different degrees of growth were selected to investigate the incidence of spinach wilt by cultivating spinach inoculated with the pathogenic *F. oxysporum* strain. A correlation (r=0.831, *P*<0.001) was observed between the disease incidence of spinach wilt and the growth degree of *F. oxysporum* using the co-cultivation method in the 14 selected soils. No correlations were noted between chemical and biological parameters (including pH and the population density of microbes, except for the growth degree of *F. oxysporum*) and the growth degree of *F. oxysporum* and incidence of spinach wilt, indicating that it was not possible to predict the degree of growth or disease incidence based on specific chemical and biological characteristics or their combination. The present results suggest that an evaluation of the growth degree of *F. oxysporum* by the *Fusarium* co-cultivation will be useful for diagnosing the disease suppressiveness of soil against pathogenic *F. oxysporum* in croplands.

Soil in which the occurrence of plant diseases is suppressed is referred to as disease suppressive soil (Baker and Cook, 1974). The chemical and biological properties of soil have been reported as factors related to the suppressiveness of soil (Kobayashi and Komada, 2011), such as soil pH (Murakami *et al.*, 2004; Niwa *et al.*, 2007), antagonistic bacteria (Innerebner *et al.*, 2011), and spore-lysing bacteria (Toyota and Kimura, 1993). Although multiple factors of soil biological properties are associated with disease suppression, not all have been confirmed and some have yet to be identified. Furthermore, since small differences in soil physicochemical properties, such as water content and soil temperature, may affect the abundance and composition of the microbial community in soil, the number of specific microorganisms and the species composition of the microbial community may not always be the same. Therefore, there is currently no established index of the soil biological properties responsible for suppressiveness. However, an evaluation of disease suppression caused by biological factors may allow effective measures against soil-borne diseases that cannot be taken based on an evaluation of soil physicochemical properties alone. While specific functional microbes, such as antagonistic bacteria (Innerebner *et al.*, 2011) and spore-lysing bacteria (Toyota and Kimura, 1993), related to disease suppression have been identified, a metagenomic ana­lysis has been applied to estimations of the functional genes or microorganisms involved in suppressiveness (Tracanna *et al.*, 2021). The microbial members involved in disease suppression have been identified by a comparative ana­lysis of a disease suppressive soil and disease conducive soil (Tracanna *et al.*, 2021). However, the degree of disease suppression caused by these microorganisms needs to be examined in various soils in order to generalize their functions. To date, specific microorganisms, such as antagonistic bacteria (Innerebner *et al.*, 2011), spore-lysing bacteria (Toyota and Kimura, 1993), and soil microflora (Tracanna *et al.*, 2021), have been assessed based the degree of disease suppression; however, few studies have comprehensively evaluated the abundance, activity, and antagonistic ability of indigenous microorganisms in the soil microbial community under crop cultivation to diagnose the suppressiveness of soil (Hashimoto *et al.*, 2008).

Among soil-borne diseases, those caused by *Fusarium* spp. affect many crop species and are difficult to control (Komada, 1984). Diseases caused by *Fusarium* spp. consist of wilt and root rot. *Fusarium oxysporum*, which causes withering in many crops, involves pathogen invasion from underground that spreads from vascular bundles to the intraductal and xylem parenchyma with a number of symptoms, including wilting, stem-end splitting, etiolation, and dry rot (Ogawa, 2011). The biological factors involved in suppressing the pathogenicity of *F. oxysporum* include the bactericidal effects of *Pseudomonas* and *Pimelobacter* against spores of *F. oxysporum* f. sp. *raphani* (Japanese radish yellow rot) (Toyota and Kimura, 1993), the antagonism of *Penicillium* against pathogenic *Fusarium* (Alam *et al.*, 2011), and the germination inhibitory effects of fungi against *F. oxysporum* f. sp. *radicis-lycopersici* (tomato root rot wilt disease) (Hamanaka *et al.*, 2005). Furthermore, difficulties are associated with evaluating the incidence of disease caused by *F. oxysporum* in actual croplands because factors that control disease may differ depending on environmental conditions. It is also challenging to individually assess all deterrent factors against pathogenic *F. oxysporum*, such as antagonistic bacteria, bacteriolytic activity, and the production of growth inhibitory substances, because different types (special forma) of pathogenic *F. oxysporum* for their respective plants may be present together in the soil. Therefore, there is currently no practical diagnostic meth­od‍ ‍to evaluate suppressiveness against pathogenic *F. oxysporum* based on the biological properties of soil.

As a soil biological diagnostic method that comprehensively evaluates the ability to suppress *F. oxysporum* pathogenicity due to the number and activity of culturable indigenous microorganisms in soil and their antagonistic ability, a co-cultivation method with soil suspensions and the pathogen (*F. oxysporum*) is being examined (*Fusarium* co-cultivation method). A correlation was observed between the incidence of spinach wilt and the growth degree of *F. oxysporum* by microorganisms in soil treated with organic fertilizers in a pot experiment inoculated with a spinach wilt disease fungus (*F. oxysporum* f. sp. *spinaciae*) (Mitsuboshi *et al.*, 2016). In addition, the suppression of *F. oxysporum* growth was demonstrated by the *Fusarium* co-cultivation method for soils in plots that showed suppressiveness against spinach wilt disease in the inoculation test of *F. oxysporum* f. sp. *spinaciae* in two experimental fields with the long-term application of organic fertilizers (Mitsuboshi *et al.*, 2018). However, the applicability of the *Fusarium* co-cultivation method has not yet been examined in croplands (farmers’ fields) in which various crop cultivations and fertilization are performed. In our previous study (Mitsuboshi *et al.*, 2018), the soils tested were collected from experimental fields with the long-term application of organic fertilizers, identical weather conditions, and the same and defined management for crop cultivation other than organic amendments continued for more than 25 years.

In the present study, we examined the possibility of evaluating soil suppressiveness against *F. oxysporum* f. sp. *Spinaciae* in soils collected from croplands across the country using the *Fusarium* co-cultivation method. We demonstrated the applicability of the *Fusarium* co-cultivation method for cropland soils using soil samples from farmers’ fields, which were located in different areas under distinct weather conditions and with various soil managements for fertilization and crop cultivation. Soils with different degrees of *F. oxysporum* growth were inoculated with *F. oxysporum* f. sp. *spinaciae*, and the relationship between the disease incidence of spinach wilt and the growth degree of *F. oxysporum* was examined. In addition, the chemical properties (such as pH, electrical conductivity [EC], and mineral nutrient content) and biological properties (population densities of microbes and enzyme activity) of soil were investigated, and the influence of these soil characteristics on the suppressiveness of soil was clarified.

## Materials and Methods

### Soil

We collected soil samples mainly from farmers’ fields located in different areas under various crop cultivation and distinct soil managements to examine the applicability of the *Fusarium* co-cultivation method to soils under these diverse conditions. Seventy-five soil samples were taken from 21 cities or counties in 11 prefectures from Akita prefecture to Kagoshima prefecture, Japan (Table 1). Among the soil samples examined, 70 were from farmers’ fields and 5 were from a long-term experimental field with the application of organic fertilizers at the Osumi Branch, Kagoshima Prefectural Institute for Agricultural Development (Kanoya, Kagoshima, Japan). These fields included open ground (upland and paddy fields) and greenhouses cropped with taro, tomato, cucumber, cherry tomato, carrot, lettuce, green onion, spinach, pumpkin, turnip, broccoli, chrysanthemum, cabbage, fig, sweet potato, and rice. Soil was collected at a depth of approximately 2 to 15‍ ‍cm between plants or stubble from 5 locations in the field with a trowel after the harvesting of crops and was mixed (approximately 500 g). Portions of soil samples for chemical ana­lyses were passed through a 2-mm sieve after air drying. The remainder of the samples for microbiological ana­lyses were stored at 4°C after removing coarse stones without sieving. The population density of microbes and enzyme activities were assessed within approximately one month after sampling. Tests for the *Fusarium* co-cultivation method and disease incidence of spinach wilt by the inoculation of *F. oxysporum* f. sp. *spinaciae* were conducted within a few months of sampling.

Data on soils from two experimental fields with the long-term application of organic fertilizers (LAOF soils) at the Togo Field, the Field Science Center, Graduate School of Bioagricultural Sciences, Nagoya University (Togo, Aichi, Japan) (5 soils) and the Tsukuba Research Institute, Katakura & Co-op Agri Corporation (Tsuchiura, Ibaraki, Japan) (5 soils) (Mitsuboshi *et al.*, 2018) were also included in the ana­lysis of the relationship between the growth degree of *F. oxysporum* and soil chemical and biological properties. The growth degree of *F. oxysporum* using the *Fusarium* co-cultivation method and the disease incidence of spinach wilt by the inoculation test in these LAOF soils have already been investigated (Mitsuboshi *et al.*, 2018).

### Pathogenic fungal strain and crop

Spinach wilt disease fungus (*F. oxysporum* f. sp. *spinaciae* MAFF 103060) and spinach (*Spinacia oleracea* L.) (“OKAME”; TAKII) were used in the present study.

### Chemical and biological characteristics of soil

Stones and coarse organic matter were removed from moist soil samples by hand. pH, EC, inorganic nutrient contents (ammonium- and nitrate-nitrogen, available phosphate by the Truog method, exchangeable potassium, exchangeable calcium, and exchangeable magnesium), the cation exchange capacity (CEC), phosphate absorption coefficient, and humus content were measured after soils were air-dried at room temperature and passed through a 2-‍mm sieve. After the reciprocal shaking of 10‍ ‍g of air-dried soil with 50‍ ‍mL of deionized water in a polyethylene container at 200‍ ‍rpm for 30‍ ‍min, soil pH and EC were measured with the pH meter M-12 (Horiba) and EC meter ES-51 (Horiba), respectively. The concentrations of elements were assessed using the Soil & Plant Analyzer SFP-4i (Fujihira Industry) following the manufacturer’s instructions for air-dried soil samples. The analyzer provides analytical data equivalent to those obtained by internationally recognized methods for soil ana­lyses based on measurements using a spectrophotometer and flame photometer (http://www.fujihira.co.jp/seihin/soi/sfp-4.html). The contents of carbon and nitrogen were evaluated using a JM1000CN MACRO CORDER (Yanaco Technical Science) following the manufacturer’s instructions for air-dried soil samples.

The population density of fungi was assessed using the dilution plate technique on rose Bengal agar medium (KH_2_PO_4_ 1 g, MgSO_4_·7H_2_O 0.5 g, peptone 5 g, glucose 10 g, rose Bengal 33‍ ‍mg, agar 17 g, streptomycin 30‍ ‍mg, and distilled water 1 L; pH 6.8) (Soil Microbiological Society of Japan, 1975). The population densities of actinomycetes and bacteria were measured using the dilution plate technique on egg albumin agar medium (egg albumin‍ ‍0.25‍ ‍g, glucose 1 g, K_2_HPO_4_ 0.5 g, MgSO_4_·7H_2_O 0.2 g, Fe_2_(SO_4_)_3_ trace, agar 15 g, and distilled water 1 L; pH 6.8) (Soil Microbiological Society of Japan, 1975). The population of *Fusarium* spp. was enumerated using the smear plate technique on Fo-G1 medium (Nishimura, 2007). β-glucosidase activity was measured by spectrophotometry (Hayano, 1973) using p-nitrophenyl β-D-glucopyranoside as the substrate. Ten grams of soil was used to assess the population densities of microorganisms (*n*=1) and 0.5‍ ‍g soil for the measurement of enzyme activity (*n*=1).

### Co-cultivation of *F. oxysporum* f. sp. *spinaciae* with soil microorganisms

Ten-gram portions of soil samples were added to a sterilized tube containing 90‍ ‍mL of sterilized tap water and shaken reciprocally at 200‍ ‍rpm for 30‍ ‍min. One milliliter of the suspension was diluted with 9‍ ‍mL of sterilized tap water, mixed well, and serially diluted in the same manner. A dilution series was prepared to a concentration of 10^–6^. One milliliter of 10^1^ to 10^6^-fold diluted suspensions was inoculated into a Petri dish and 16‍ ‍mL of YPMG agar medium (Peptone-yeast extract medium; yeast extract 3 g, peptone 5 g, beef extract 3 g, glucose 10 g, agar 15 g, and distilled water 1 L; pH 7.0) (Soil Microbiological Society of Japan, 1975) was poured and mixed. A square section of the *F. oxysporum* hyphal lawn was placed at the center of the agar medium. As a control, sterilized water was inoculated instead of the diluted soil sample suspension. Plates were incubated at 30°C for approximately one week (7 or 8 d), by which time the *F. oxysporum* colony had spread fully on the control plate. The length of the shortest part of the colony together with the longest part was measured; *i.e.*, the extension of hyphae was measured at parts where the hyphae had grown the most (long diameter) and the least (short diameter) in the *F. oxysporum* colony for soil samples and the control, and the mean of these values was used to calculate the degree of growth. As a representative value for the growth degree of *F. oxysporum*, the median of the estimated values of the growth degree at six dilutions from 10^–1^ to 10^–6^ was calculated (Mitsuboshi *et al.*, 2016).

### Cultivation of crops and inoculation of the pathogenic *F. oxysporum* strain

The cultivation of crops and inoculation of *F. oxysporum* f. sp. *spinaciae* was performed with 14 soil samples differing in the growth degree of *F. oxysporum* using the *Fusarium* co-cultivation method. The 14 soil samples were selected based on differences in the growth degrees of *F. oxysporum* by the *Fusarium* co-cultivation method, as described in the Results section. Spinach seeds pretreated in water for 2‍ ‍d were sown into a cell tray (200 holes) filled with nursery soil (“YASAI-BAIDO ICHI GOU”; Katakura & Co-op Agri Corporation) and grown in a greenhouse for 10–14 d. Conidiospores of *F. oxysporum* f. sp. *spinaciae* were added to soil samples at a dose of 10^6^ conidia g^–1^ soil and 500‍ ‍mL of soil (approximately 400 g) was placed in polycarbonate pots (12‍ ‍cm outer diameter×11.5‍ ‍cm height; 0.01 m^2^). To achieve the proliferation of conidiospores, *F. oxysporum* was cultivated on potato dextrose agar (potato extract [prepared from 1‍ ‍kg potatoes boiled in 1‍ ‍L of water] 100‍ ‍mL, glucose 20 g, agar 15 g, and distilled water 900‍ ‍mL) at 30°C for 7‍ ‍d and a square section of the fungal lawn with 5‍ ‍mm on each side was cultured in 100‍ ‍mL of potato sucrose broth (potato extract 100‍ ‍mL, sucrose 20 g, and distilled water 900‍ ‍mL) at 30°C for 7‍ ‍d by shaking horizontally. The number of conidiospores was enumerated on a hemocytometer and diluted culture solutions with a predetermined density were used for the inoculation. Soils were fertilized with a compound inorganic fertilizer (N-P-K=125-250-125‍ ‍mg pot^–1^, equivalent to N-P-K=​150-300-150‍ ‍kg ha^–1^ assuming that the bulk density of soil was 1.0 and the depth of the plow layer was 0.1 m), inoculated with conidiospores, and planted with spinach seedlings on the same day. Three cell seedlings of spinach were transplanted in each pot with 3 or 4 replicates, and disease incidence was investigated after cultivation for a certain period of time. Inoculation tests were performed in four different periods as follows: seeding on May 12, 2016, February 24, 2017, March 14, 2017, and March 21, 2017, transplanting on May 23, 2016 (11‍ ‍d after seeding), March 10, 2017 (14‍ ‍d after seeding), March 24, 2017 (10‍ ‍d after seeding), and March 31, 2017 (10‍ ‍d after seeding). We noted the disease incidence and severity of wilting for each spinach plant on June 6, 2016 (14‍ ‍d after planting), March 30, 2017 (20‍ ‍d after planting), April 11, 2017 (18‍ ‍d after planting), and April 18, 2017 (18‍ ‍d after planting). The incidence and severity of wilt were evaluated as follows: 0, healthy; 1, one leaf had wilted; 2, two or three leaves had wilted; 3, half of the leaves had wilted; 4, more than half of the leaves had wilted; 5, dead or nearly dead. Soil from the inorganic fertilizer plot (a disease conducive soil; Cont) in the experimental field with the long-term application of organic fertilizers at the Tsukuba Research Institute, Katakura & Co-op Agri Corporation was used to evaluate differences in the growth of spinach and disease incidence among the different growing periods of spinach. During the cultivation and inoculation test, the moisture content of soil was maintained by watering to maintain appropriate conditions for the growth of spinach and disease incidence.

### Statistical ana­lysis

All statistical tests were performed with Microsoft Excel 2016 for Windows (Microsoft) and BellCurve for Excel version 2.12 (Social Survey Research Information). Differences in the disease incidence of spinach from those in control pots were statistically tested using the Steel test. The degree of *F. oxysporum* growth at each dilution stage of each soil was tested for differences by the Tukey-Kramer method. The relationship between the disease incidence of spinach wilt and the growth degree of *F. oxysporum* estimated by the co-cultivation method was analyzed by Spearman’s rho. A cluster ana­lysis (variable classification) was performed by the Ward method to classify soil chemical and biological variables. The relationships between the chemical and biological properties of soil, *F. oxysporum* growth, and disease incidence by the pathogen inoculation test were analyzed by non-metric multi-dimensional scaling, a principal component ana­lysis, and multiple regression ana­lysis.

## Results

### Growth degree of *F. oxysporum* by the *Fusarium* co-cultivation method for soil

**Fig. 1.**
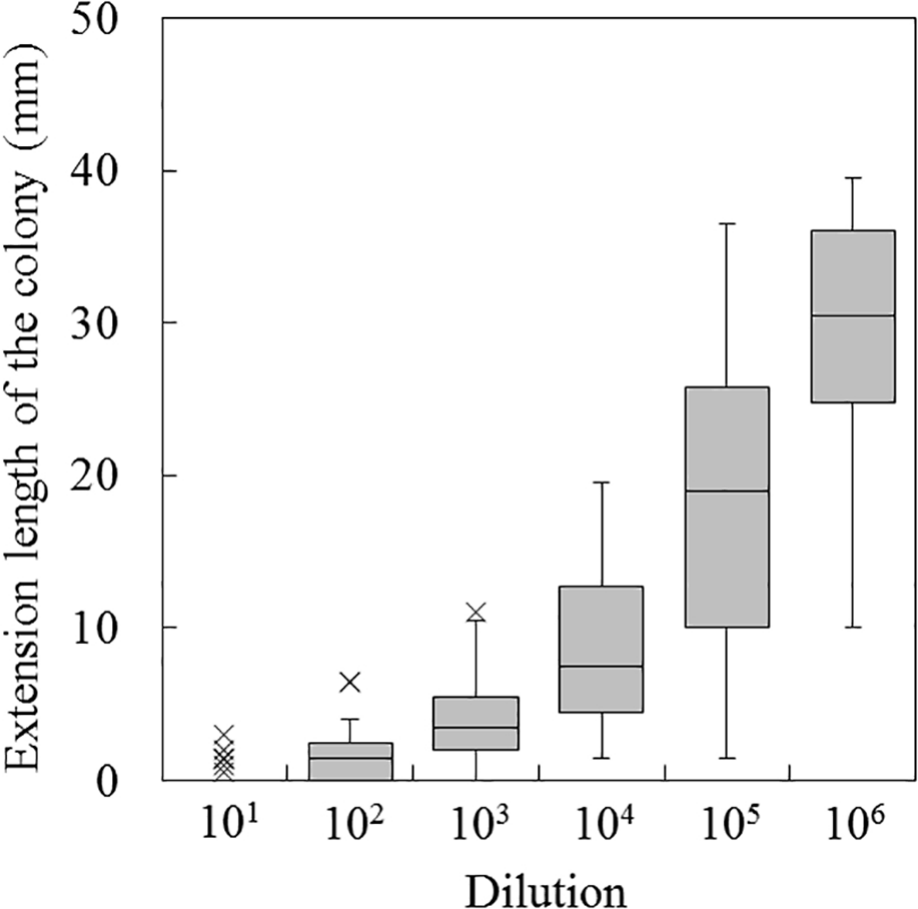
Growth degree of *Fusarium oxysporum* f. sp. *spinaciae* by the *Fusarium* co-cultivation method (mm) for soil samples from croplands (*n*=75). The extension length for control plates ranged between 36.5 and 40.5‍ ‍mm. The box indicates the first quartile to the third quartile with the median value, the whisker indicates 1.5 times the range of the interquartile, and x indicates the outlier value (a value greater than 1.5 times the range of the interquartile).

The growth degree of *F. oxysporum* at every dilution stage of 75 soil samples gradually increased at higher dilutions (Fig. 1). Among 75 soil samples, 68 showed a *F. oxysporum* growth degree of 0‍ ‍mm at the 10^–1^ dilution, whereas 7 showed 0.5–3‍ ‍mm. In the soil suspension at 10^–3^ dilution, a soil sample showed the suppression of *F. oxysporum* growth with a growth degree of 0‍ ‍mm; however, there was no soil sample with a growth degree of 0‍ ‍mm at the 10^–4^ dilution. The largest difference in the magnitude of the growth degree of *F. oxysporum* was observed at the 10^–5^ dilution. Since the maximum growth degree of *F. oxysporum* was approximately 40‍ ‍mm from the size of the plate, a plate showing 40‍ ‍mm at the 10^–6^ dilution indicated that the growth of *F. oxysporum* was not suppressed. The growth degrees of *F. oxysporum* at each dilution stage of six soil samples were significantly lower than those from Kanoya 2 in Kagoshima prefecture (mineral fertilizer plot in‍ ‍Osumi Branch, Kagoshima Prefectural Institute for Agri­cultural Development), which showed the highest growth‍ ‍degrees. In contrast, soil samples from Yokote in Akita prefecture, Fukushima in Fukushima prefecture, Kawachi in Tochigi prefecture, Bando in Ibaraki prefecture, and Tsuchiura in Ibaraki prefecture exhibited relatively low growth degrees and the growth degrees of several soil samples (1 to 9 for the respective soil samples) were significantly higher than those of the five soil samples (the Tukey-Kramer method). The median value of the growth degree of‍ ‍*F. oxysporum* was in the range of 1.0 to 13.8‍ ‍mm, and the‍ ‍average value was 6.3‍ ‍mm. The growth degree of *F.‍ ‍oxysporum* was less than 5.0‍ ‍mm in 45% of all samples, and the number of samples decreased as the value increased in soil samples of 5.0‍ ‍mm or more (Fig. S1).

### Disease incidence of spinach wilt by the inoculation of *F. oxysporum* f. sp. *spinaciae* and the growth degree of *F. oxysporum* for selected soil samples

**Fig. 2.**
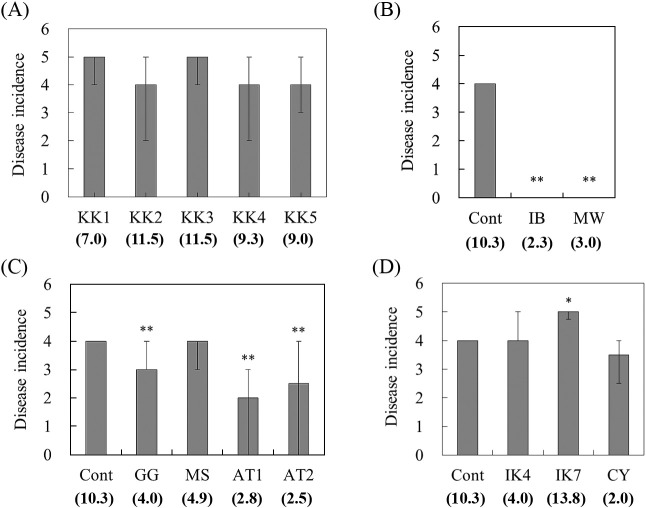
Disease incidence of spinach wilt by *Fusarium oxysporum* f. sp. *spinaciae* for 14 selected soil samples from croplands. The values in parentheses show the median values of growth degrees of *F. oxysporum* f. sp. *spinaciae* by the *Fusarium* co-cultivation method (mm). A disease conducive soil (inorganic fertilizer plot at the Tsukuba Research Institute Farm, Katakura & Co-op Agri Corporation) was used as the control (Cont) for comparison. Inoculation tests were performed in four different seeding periods (A, May 23, 2016; B, February 24, 2017; C, March 14, 2017; D, March 21, 2017). KK, Kanoya, Kagoshima; IB, Bando, Ibaraki; MW, Watari, Miyagi; GG, Gifu, Gifu; MS, Suzuka, Mie; AT, Tahara, Aichi; IK, Komatsu, Ishikawa; CY, Yachimata, Chiba. Values show the medians of disease incidence with the first and third quartiles. (*n*=9, A; *n*=12, B, C, and D). * and ** indicate significant differences from the Cont (B, C, and D) at the 5% and 1% levels (*P*<0.05, and *P*<0.01; the Steel test), respectively.

**Fig. 3.**
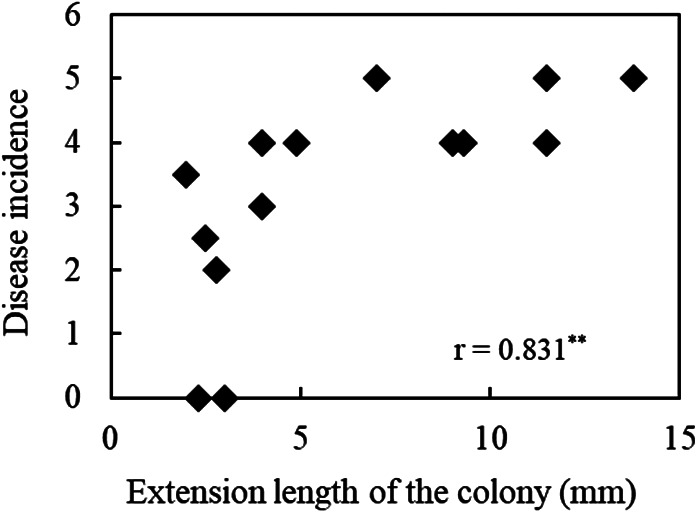
Relationship between median values of growth degrees of *Fusarium oxysporum* f. sp. *spinaciae* by the *Fusarium* co-cultivation method (mm) and disease incidence for selected soil samples from croplands (*n*=14). ** indicates a significant difference (*P*<0.01, Spearman’s rho).

Inoculation experiments of the pathogenic *F. oxysporum* strain were performed for 14 soil samples showing different growth degrees of *F. oxysporum* by the *Fusarium* co-cultivation method as follows: 3 soil samples with the representative growth degrees of *F. oxysporum* ranging between 0 and 2.5‍ ‍mm, 5 soil samples with growth degrees between 2.5 and 5.0‍ ‍mm, 1 soil sample with growth degrees between 5.0 and 7.5‍ ‍mm, 2 soil samples with growth degrees between 7.5 and 10.0‍ ‍mm, 2 soil samples with growth degrees between 10.0 and 12.5‍ ‍mm, and 1 soil sample with growth degrees of 12.5‍ ‍mm or more. The Cont soil (a disease conducive soil) showed a disease incidence of 4, as previously reported (Mitsuboshi *et al.*, 2018). Nine out of 14 soil samples showed a disease incidence of 4 to 5, and one (IK 7, Komatsu in Ishikawa prefecture) had a significantly higher disease incidence than conducive soil (Cont) (*P*<0.05). In addition, the disease incidence was significantly lower on 5 soil samples (*P*<0.01) than on conducive soil (Cont) (Fig. 2). In the relationship between the median value of growth degrees by the *Fusarium* co-cultivation method and the incidence of spinach wilt for the 14 soil samples, soil with a high growth degree of *F. oxysporum* showed a high disease incidence of 4 to 5, while soil with a growth degree of *F. oxysporum* less than 4‍ ‍mm showed a low disease incidence of 4 and less than 4. A positive correlation (*r*=0.831; *P*<0.001, Spearman’s rho) was observed between the growth degree of *F. oxysporum* and disease incidence (Fig. 3).

### Relationship between growth degrees of *Fusarium oxysporum* and soil chemical and biological properties

The chemical and biological properties of soil samples are shown in Table S1. To clarify the effects of various soil characteristics on the growth degree of *F. oxysporum* and disease incidence, the chemical and biological (excluding the growth degree of *F. oxysporum*) properties of 14 soil samples were examined by the non-metric multi-dimensional scaling method, principal component ana­lysis, and multiple regression ana­lysis.

In the non-metric multi-dimensional scaling method, there was no tendency of orderly plotting according to the magnitude of the growth degree of *F. oxysporum* (Fig. S2A). Regarding the disease incidence, samples with low and high values were plotted on both the plus and minus sides in dimension 1, indicating no tendency of plotting due to the difference in the disease incidence (Fig. S2B). Therefore, no clear relationship between soil chemical and biological properties and the growth degree of *F. oxysporum* or disease incidence was observed.

**Fig. 4.**
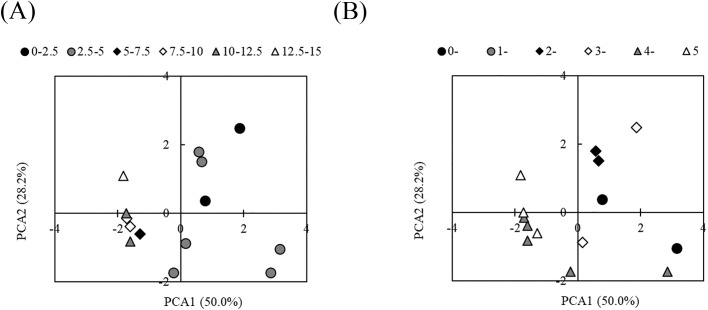
Ordination of 14 selected soil samples from croplands based on chemical and biological characteristics by a principal component ana­lysis. pH, EC, the exchangeable Ca content, humus content, population density of fungi, and β-glucosidase activity were used. The legend shows the median values of growth degrees of *Fusarium oxysporum* f. sp. *spinaciae* by the *Fusarium* co-cultivation method (mm) (A) and the disease incidence of spinach wilt by *F. oxysporum* f. sp. *spinaciae* (B) (*n*=14).

The principal component ana­lysis was performed by excluding variables with strong correlations to avoid multicollinearity. A cluster ana­lysis (variable classification) of group variables with high similarity was conducted using data from 85 soil samples, including LAOF soil samples from two experimental fields with the long-term application of organic fertilizers at the Togo Field, the Field Science Center, Graduate School of Bioagricultural Sciences, Nagoya University and the Tsukuba Research Institute, Katakura & Co-op Agri Corporation (Fig. S3). Based on a dissimilarity value of 1.45, six properties from the six clusters were selected as explanatory variables. Depending on the difference in the growth degree of *F. oxysporum*, samples with growth degrees between 0 and 5‍ ‍mm were plotted on the plus side of the first component, whereas samples with growth degrees of 5‍ ‍mm or more were more likely to be plotted on the minus side (Fig. 4A). The contribution ratios of the first and second components were 50 and 28.2%, respectively. The loading values of the first principal component were in the order of 0.903, 0.700, 0.666, 0.589, –0.625, and –0.716 for EC, the population density of fungi, β-glucosidase activity, the exchangeable Ca content, pH, and the humus content, respectively. The scores of each explanatory variable as vectors for the first and second principal components were 0.522 and 0.233, 0.404 and –0.262, 0.384 and –0.512, 0.340 and 0.588, –0.361 and 0.413, and –‍0.413 and –0.315 for EC, the population density of fungi, β-glucosidase activity, the exchangeable Ca content, pH, and the humus content, respectively. However, there was no clear trend in the plot for the principal component ana­lysis of disease incidence (Fig. 4B).

In the multiple regression ana­lysis (stepwise method), it was necessary to reduce the number of explanatory variables because the number of soil samples was smaller than the number of variables. According to correlation coefficients between variables in the same cluster by the cluster ana­lysis (variable classification; Fig. S3), the inorganic nitrogen content, exchangeable Mg content, and CEC were excluded from the ana­lysis. Table 2 shows standardized partial regression coefficients and P-values for the multiple regression ana­lysis using the growth degree of *F. oxysporum* as the objective variable. The regression equation showed significance (*P*<0.001). The population densities of fungi and *Fusarium* spp., and the exchangeable Ca content affected the direction in which the growth degree of *F. oxysporum* decreased; the exchangeable K content and the phosphate absorption coefficient influences the direction in which the growth degree of *F. oxysporum* increased. In the multiple regression ana­lysis (stepwise method) with disease incidence as an objective variable, no explanatory variable was removed in the process of variable selection and the regression equation was significant (*P*<0.001); however, there was no significant partial regression coefficient (Table 3).

In addition, non-metric multi-dimensional scaling and the principal component ana­lysis were performed using soil chemical and biological properties, excluding the growth degree of *F. oxysporum*, in 85 soils, including the 10 LAOF soils (Mitsuboshi *et al.*, 2018). The plots by these ana­lyses were arranged as scattered throughout irrespective of the difference in the growth degree of *F. oxysporum*, indicating that the relationships between soil chemical and biological properties and the growth degree of *F. oxysporum* were unclear (Fig. S4). A multiple regression ana­lysis (stepwise method) was conducted with the growth degree of *F. oxysporum* as the objective variable and soil chemical and biological properties, excluding the growth degree of *F. oxysporum*, as explanatory variables. The multiple correlation coefficient was 0.730 and the regression equation showed significance (*P*<0.001). The standardized partial regression coefficient was significant for the population density of fungi (–0.412), the exchangeable Ca content (–‍0.646), pH (0.310), and CEC (0.566) at *P*<0.001, *P*<0.01, *P*<0.01, and *P*<0.05, respectively (Table 4).

## Discussion

The growth degree estimated by the *Fusarium* co-cultivation method varied at every dilution stage for 75 soil samples (Fig. 1). Some soils showed a degree of approximately 40‍ ‍mm, the upper limit of the growth degree of *F. oxysporum* on the plate at 10^–6^ dilution, indicating that the dilution was too high to accurately compare suppressiveness among soils due to a smaller variation in the growth degree of *F. oxysporum* at 10^–6^ dilution than at 10^–5^ dilution. Colonies of microorganisms on the plate at 10^–4^ dilution may have mainly suppressed the growth degree of *F. oxysporum* to approximately 20‍ ‍mm. At 10^–5^ dilution, the growth of *F. oxysporum* was not completely suppressed by the microorganisms in the soil suspension, resulting in the greater proliferation of *F. oxysporum* with large variations than at 10^–4^ dilution. Therefore, the dilution stage at 10^–5^ is considered to be suitable to observe a difference in the suppression of the growth degree of *F. oxysporum* between soils. The growth degree of *F. oxysporum* gradually increased as the soil dilution ratio increased in the pot experiment applied with organic fertilizers to soil (Mitsuboshi *et al.*, 2016); however, the growth of *F. oxysporum* was suppressed even at 10^–6^ dilution in some soils. On the other hand, variations in the growth degree of *F. oxysporum* were observed at the 10^–5^ dilution for LAOF soils that exhibited suppression against spinach wilt disease and the growth degree of *F. oxysporum* was approximately 40‍ ‍mm in some soils at 10^–6^ dilution (Mitsuboshi *et al.*, 2018). The 75 soils examined in the present study showed similar changes in the growth degree of *F. oxysporum* at 10^–5^ and 10^–6^ dilution stages as that of the LAOF soils (Mitsuboshi *et al.*, 2018). A significant difference in the growth degree of *F. oxysporum* at each dilution stage was also observed among some soil samples from croplands under diverse field management, such as fertilization. These results indicate that the *Fusarium* co-cultivation method is also applicable to soils in the croplands by evaluating the difference in the growth degree of *F. oxysporum* caused by the indigenous microorganisms in the soils.

The median value of the growth degree of *F. oxysporum* was less than 5‍ ‍mm in 45% of all soil samples. In a previous study (Mitsuboshi *et al.*, 2016), the growth degree was less than 5‍ ‍mm in 20% of samples (Mitsuboshi *et al.*, 2016), while in a study on LAOF soils, the growth degree of soil without the inoculation of pathogenic *F. oxysporum* was less than 5‍ ‍mm in 10% of samples (Mitsuboshi *et al.*, 2018). In the croplands used in the present study, a growth degree of *F. oxysporum* of less than 5‍ ‍mm was observed in a larger percentage (45%) of samples than in previous studies. In contrast to the experimental fields with defined fertilization, many types of fertilizers and amendments may be applied to the soil in croplands under various field managements. These diverse conditions of soils in the croplands may have influenced the difference in the percentages of samples showing a growth degree of *F. oxysporum* of less than 5‍ ‍mm from the soils in pot experiments (Mitsuboshi *et al.*, 2016) and LAOF soils (Mitsuboshi *et al.*, 2018) in previous studies.

A positive correlation between the growth degree of *F. oxysporum* and disease incidence was also shown for the soil in croplands, similar to the pot experiment with the application of organic fertilizers to a soil (Mitsuboshi *et al.*, 2016) and in the study of experimental fields with the long-term application of organic fertilizers (Mitsuboshi *et al.*, 2018). Therefore, suppressiveness against spinach wilt disease may be evaluated for the soil in croplands by measuring the growth degree of *F. oxysporum* using the *Fusarium* co-cultivation method. The disease incidence was mostly maintained at between 0 and 3 even when the growth degree of *F. oxysporum* was between 5 and 10‍ ‍mm and samples with *F. oxysporum* growth less than 5‍ ‍mm did not show a high degree of disease incidence in the pot experiment (Mitsuboshi *et al.*, 2016) or in the study of experimental fields (LAOF soils) (Mitsuboshi *et al.*, 2018). In the present study, soils with a *F. oxysporum* growth degree of 4‍ ‍mm or more showed a disease incidence of 4 or higher, whereas soils with a growth degree of *Fusarium* less than 4‍ ‍mm did not necessarily suppress the disease incidence. In previous studies (Mitsuboshi *et al.*, 2016, 2018), microorganisms in organic fertilizers that were applied to the soils may have been involved in suppressing the growth of *F. oxysporum* in the co-cultivation method and decreasing the disease incidence of spinach wilt in the inoculation tests. However, the conditions may be complex in the soil of croplands; some soils with a small growth degree of *F. oxysporum* did not suppress the disease incidence in the pathogen inoculation test. Some factors other than the growth degree of *F. oxysporum*, such as soil chemical properties, affected the disease incidence of the soil from croplands in the inoculation test. It is important to note that the disease incidence may differ in some cases even if the growth degree of *F. oxysporum* is small in the evaluation by the *Fusarium* co-cultivation method for the soil of croplands. In our previous study (Mitsuboshi *et al.*, 2018), no correlations were observed between the suppressiveness of soils in the experimental field with the long-term application of organic fertilizers and bacterial diversity based on amplicon sequencing of the 16S rRNA gene. However, it is possible that microorganisms that did not grow on the medium used in the *Fusarium* co-cultivation method were involved in suppressiveness (Jayaraman *et al.*, 2021). Further studies are warranted to investigate the functions of these microorganisms related to suppressiveness.

Non-metric multi-dimensional scaling did not show clear relationships between the chemical and biological properties of soils and the growth degree of *F. oxysporum* in the ana­lysis of the 14 selected soil samples (Fig. S2A). On the other hand, the principal component ana­lysis of the 14 soil samples using the 6 variables selected from soil chemical and biological properties showed that high values for EC, the population density of fungi, β-glucosidase activity, and the exchangeable Ca content slightly decreased the growth degree of *F. oxysporum* (Fig. 4). A multiple regression ana­lysis indicated high values for the population densities of fungi and *Fusarium* sp., and the exchangeable Ca content suppressed the growth degree of *F. oxysporum* (Table 2), which was consistent with the results of the principal component ana­lysis. However, non-metric multi-dimensional scaling and the principal component ana­lysis did not show clear relationships between the chemical and biological properties of soils and the growth degree of *F. oxysporum* in the ana­lysis of 85 soil samples (Fig. S4). The multiple regression ana­lysis identified chemical and biological properties that positively or negatively influenced the growth degree of *F. oxysporum* in the 85 soils (Table 4); however, the set of variables were not identical with those for the 14 selected soils (Table 2). These inconsistencies in the ana­lysis using the 85 and 14 soils suggested that specific chemical and biological properties or a combination of these properties involved in the suppression of *F. oxysporum* growth differed between the 85 and 14 soils. If the effects of any chemical or biological properties on the suppression of the growth degree of *F. oxysporum* were consistent in soils, the results of these ana­lyses may be similar regardless of whether the 14 or 85 soil samples were used. However, this was not the case. These results indicate that the relationship between soil chemical and biological properties and the growth degree of *F. oxysporum* by the *Fusarium* co-cultivation method vary depending on the soils in croplands. In contrast, no clear relationship between disease incidence and the chemical and biological properties of soil was observed in non-metric multi-dimensional scaling (Fig. S2B), the principal component ana­lysis (Fig. 4B), or multiple regression ana­lysis (Table 3) of the 14 selected soil samples. Disease incidence was not clearly explained by the combination of the chemical and biological properties of soil in the croplands. Consequently, to evaluate the disease suppressiveness of cropland soils, an estimation of the growth degree of *F. oxysporum* by the *Fusarium* co-cultivation method is necessary since no clear relationship was found between the chemical and biological properties of soils and the growth degree of *F. oxysporum* by the co-cultivation method.

In a literature review by Bonanomi *et al.* (2010), multiple properties, such as fluorescein diacetate (FDA) hydrolysis activity, respiratory activity, microbial biomass, the bacterial number, and *Trichoderma* density in soil were involved in the suppression of soil-borne diseases caused by the application of organic matter, indicating no specific factor for suppressiveness. According to Weller *et al.* (2002), it is necessary to clarify the microbial community structure of suppressive soil and then define how the activities of important microbial communities are involved in suppressiveness. The characterization of many parameters is necessary for an evaluation of suppressiveness, such as the soil structure and composition, suitable environmental conditions for suppressiveness, interactions among important microbial communities under different conditions, and the population dynamics of microbial communities as well as pathogens; however, this is practically impossible. In contrast, antagonistic microorganisms against pathogens in soil were found to be increased by root secretions when the same crop was continuously cultivated (Cha *et al.*, 2016). Although antagonistic microorganisms against pathogens may be involved in the suppressiveness of soil formed by the application of org­anic‍ ‍matter or compost, the application of organic matter or compost does not necessarily result in suppressiveness and‍ ‍the suppressive effects of soil microorganisms cannot‍ ‍be‍ ‍expected at all times (Jambhulkar *et al.*, 2015). These findings also highlight the difficulties associated with defining a common index of soil properties to evaluate the suppressiveness of soil, although specific chemical or biological properties of soil may have affected the suppression of soil-borne diseases in some soils. Overall, the suppressiveness of soil against pathogenic *F. oxysporum* cannot be predicted based on the chemical and biological properties of soils in croplands. Therefore, it is possible to estimate the growth degree of *F. oxysporum* by the *Fusarium* co-cultivation method for each soil in order to evaluate suppressiveness, where the growth degree of *F. oxysporum* is generally difficult to explain by the combination of the chemical and biological properties of soil in the croplands.

## Conclusion

Differences in the growth degree of *F. oxysporum* f. sp. *spinaciae* by the *Fusarium* co-cultivation method were found for 75 soils collected from croplands around the country and a correlation was observed between the disease incidence of spinach wilt and the growth degree of *F. oxysporum*. The chemical and biological properties of soil affecting the growth degree of *F. oxysporum* by the co-cultivation method and the disease incidence of soil-borne disease were not clear and a common index to predict the growth degree of *F. oxysporum* or the disease incidence for the soils in croplands was not defined. Therefore, by evaluating the growth degree of *F. oxysporum*, the *Fusarium* co-cultivation method may be a useful tool for diagnosing the suppressiveness of soil against spinach wilt disease in croplands.

## Citation

Mitsuboshi, M., Kioka, Y., Noguchi, K., and Asakawa, S. (2022) Evaluation of Disease Suppressiveness of Soils in Croplands by Co-Cultivation of Pathogenic *Fusarium oxysporum* and Indigenous Soil Microorganisms. *Microbes Environ ***37**: ME21063.

https://doi.org/10.1264/jsme2.ME21063

## Supplementary Material

Supplementary Material

## Figures and Tables

**Table 1.** Location of croplands used for the collection of soil samples, cultivated crops, and climate

Place*	Crop	Field	No. of samples	Annual precipitation (mm)	Annual average temperature (°C)
Yokote, Akita (39.3112, 140.5531)	Taro	Open ground (upland field)	3	2,094	11.0
Watari, Miyagi (38.0441, 140.8675)	Tomato	Greenhouse	2	1,339	12.3
Fukushima, Fukushima (37.7608, 140.4747)	Cucumber	Greenhouse	4	1,203	13.4
Kawachi, Tochigi (36.4393, 139.9098)	Cherry tomato	Greenhouse	1	1,308	14.1
Ishioka, Ibaraki (36.1908, 140.2872)	Carrot	Open ground (upland field)	1	1,164	14.7
Omitama, Ibaraki (36.2392, 140.3525)	Carrot	Open ground (upland field)	1	1,164	14.7
Tsukuba Mirai, Ibaraki (35.9630, 140.0370)	Carrot	Open ground (upland field)	1	1,201	14.2
Tsuchiura, Ibaraki (36.0783, 140.2046)	Cucumber	Greenhouse	4	1,171	14.7
Tsuchiura, Ibaraki (36.0783, 140.2046)	Carrot	Open ground (upland field)	1	1,171	14.7
Namegata, Ibaraki (35.9905, 140.4890)	Carrot	Open ground (upland field)	1	1,305	13.6
Bando, Ibaraki (36.0484, 139.8887)	Lettuce	Open ground (upland field)	2	1,067	14.3
Bando, Ibaraki (36.0484, 139.8887)	Green onion	Open ground (upland field)	2	1,067	14.3
Yuki, Ibaraki (36.2896, 139.8715)	Cherry tomato	Greenhouse	1	1,083	14.7
Yuki, Ibaraki (36.2896, 139.8715)	Carrot	Open ground (upland field)	1	1,083	14.7
Asahi, Chiba (35.7204, 140.6465)	Carrot	Open ground (upland field)	1	1,489	15.3
Yachimata, Chiba (35.6658, 140.3179)	Tomato	Greenhouse	4	1,414	14.7
Katori, Chiba (35.8977, 140.4991)	Spinach	Greenhouse	1	1,447	14.2
Sanbu, Chiba (35.6029, 140.4135)	Cucumber	Greenhouse	4	1,704	15.9
Komatsu, Ishikawa (36.4083, 136.4455)	Rice	Open ground (paddy field)	8	2,253	14.4
Takayama, Gifu (36.1460, 137.2521)	Pumpkin	Open ground (upland field)	5	1,669	11.1
Takayama, Gifu (36.1460, 137.2521)	Turnip	Open ground (upland field)	5	1,669	11.1
Gifu, Gifu (35.4232, 136.7607)	Broccoli	Open ground (upland field)	5	1,864	15.9
Tahara, Aichi (34.6687, 137.2642)	Chrysanthemum	Greenhouse	5	1,680	16.2
Tahara, Aichi (34.6687, 137.2642)	Cabbage	Open ground (upland field)	1	1,680	16.2
Matsuzaka, Mie (34.5779, 136.5275)	Rice	Open ground (paddy field)	1	1,680	16.1
Suzuka, Mie (34.8819, 136.5841)	Fig	Open ground (upland field)	5	1,831	14.9
Kanoya, Kagoshima^#^ (31.3782, 130.8522)	Sweet potato	Open ground (upland field)	5	2,930	17.2

* The north latitude and east longitude of the city/town office are shown in parentheses. ^#^ Long-term experimental fields with the application of organic fertilizers at the Osumi Branch, Kagoshima Prefectural Institute for Agricultural Development, Kanoya, Kagoshima, Japan.

**Table 2.** Multiple linear regression ana­lysis (stepwise method) of median values of growth degrees of *Fusarium oxysporum* f. sp. *spinaciae* as the objective variable for selected soil samples from croplands (*n*=14)

Variable	Partial regression coefficient	Standardized partial regression coefficient	*P* value	
Fungi	–4.67	–0.648	0.001	**
*Fusarium* spp.	–2.77	–0.596	0.001	**
Exchangeable K	3.98×10^–2^	0.532	0.030	*
Exchangeable Ca	–2.35×10^–2^	–0.939	<0.001	**
Phosphate absorption coefficient	4.88×10^–3^	0.800	0.002	**
Constant	35.8		<0.001	**

Objective variable, growth degree of *Fusarium oxysporum* f. sp. *spinaciae*; explanatory variables, population densities of fungi, actinomycetes, bacteria, and *Fusarium* spp., pH, EC, available phosphate content, exchangeable K content, exchangeable Ca content, humus content, phosphate absorption coefficient, and β-glucosidase activity. The regression equation is significant at *P*<0.001 (*n*=14). * and ** indicate significant differences at the 5% and 1% levels (*P*<0.05 and *P*<0.01), respectively.

**Table 3.** Multiple linear regression ana­lysis (stepwise method) of the disease incidence of spinach wilt as the objective variable for selected soil samples from croplands (*n*=14)

Variable	Partial regression coefficient	Standardized partial regression coefficient	*P* value
Fungi	13.30	4.57	0.091
Actinomycetes	–57.00	–12.20	0.080
Bacteria	11.00	3.12	0.104
*Fusarium* spp.	–3.83	–2.04	0.093
pH	–28.90	–6.95	0.086
EC	–136.00	–21.00	0.083
Exchangeable K	0.431	14.30	0.084
Exchangeable Ca	7.63×10^–2^	7.57	0.078
Humus	–5.52	–12.90	0.088
Phosphate absorption coefficient	2.47×10^-–2^	10.00	0.088
β-glucosidase activity	–3.68×10^–2^	–4.83	0.087
Available P	–135×10^–2^	–0.99	0.171
Constant	443.00		0.077

Objective variable, the disease incidence of spinach wilt; explanatory variables, population densities of fungi, actinomycetes, bacteria, and *Fusarium* spp., pH, EC, exchangeable K content, exchangeable Ca content, humus content, phosphate absorption coefficient, β-glucosidase activity, and available P content. The regression equation is significant at *P*<0.001 (*n*=14).

**Table 4.** Multiple linear regression ana­lysis (stepwise method) of median values of growth degrees of *Fusarium oxysporum* f. sp. *spinaciae* as the objective variable for soil samples from croplands (*n*=75) and experimental fields with the long-term application of organic fertilizers (Mitsuboshi *et al.*, 2018) (*n*=10)

Variable	Partial regression coefficient	Standardized partial regression coefficient	*P* value	
Fungi	–3.20	–0.412	<0.001	**
Actinomycetes	–1.93	–0.178	0.0910	
Bacteria	–1.84	–0.167	0.0886	
pH	1.96	0.310	0.0053	**
Available P	5.40×10^–3^	0.216	0.0602	
Exchangeable K	9.10×10^–3^	0.164	0.1153	
Exchangeable Ca	–1.18×10^–2^	–0.646	0.0088	**
Exchangeable Mg	–1.87×10^–2^	–0.275	0.1157	
Humus	–0.293	–0.200	0.1045	
CEC	0.267	0.566	0.0135	*
Constant	38.10		<0.001	**

Objective variable, the growth degree of *Fusarium oxysporum* f. sp. *spinaciae*; explanatory variables, population densities of fungi, actinomycetes, and bacteria, pH, available P content, exchangeable K content, exchangeable Ca content, exchangeable Mg content, humus content, and CEC. The regression equation is significant at *P*<0.001 (*n*=85). * and ** indicate significant differences at the 5% and 1% levels (*P*<0.05 and *P*<0.01), respectively.
