# Supplementary material for: Evaluation of Disease Suppressiveness of Soils in Croplands by Co-Cultivation of Pathogenic Fusarium oxysporum and Indigenous Soil Microorganisms
Source: Microbes Environ. 2022 Oct 1;37(4):ME21063. doi: 10.1264/jsme2.ME21063 (PMC9763048; doi:10.1264/jsme2.ME21063)
Supplement: Supplementary file 1 — Supplementary Material [file 37_21063_s1.pdf]

Fig. S1

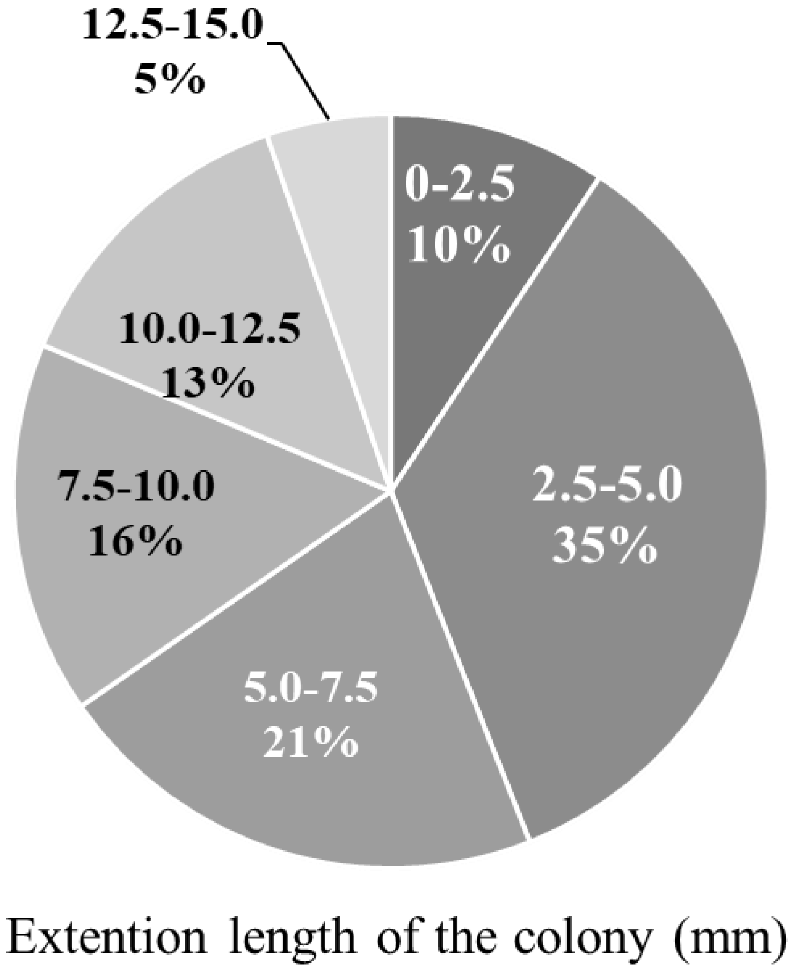

Fig. S1. Distribution of median values of growth degrees of *Fusarium oxysporum* f. sp. *spinaciae* by the *Fusarium* co-cultivation method at 2.5 mm intervals for the soil samples from croplands (n = 75).

Fig. S2

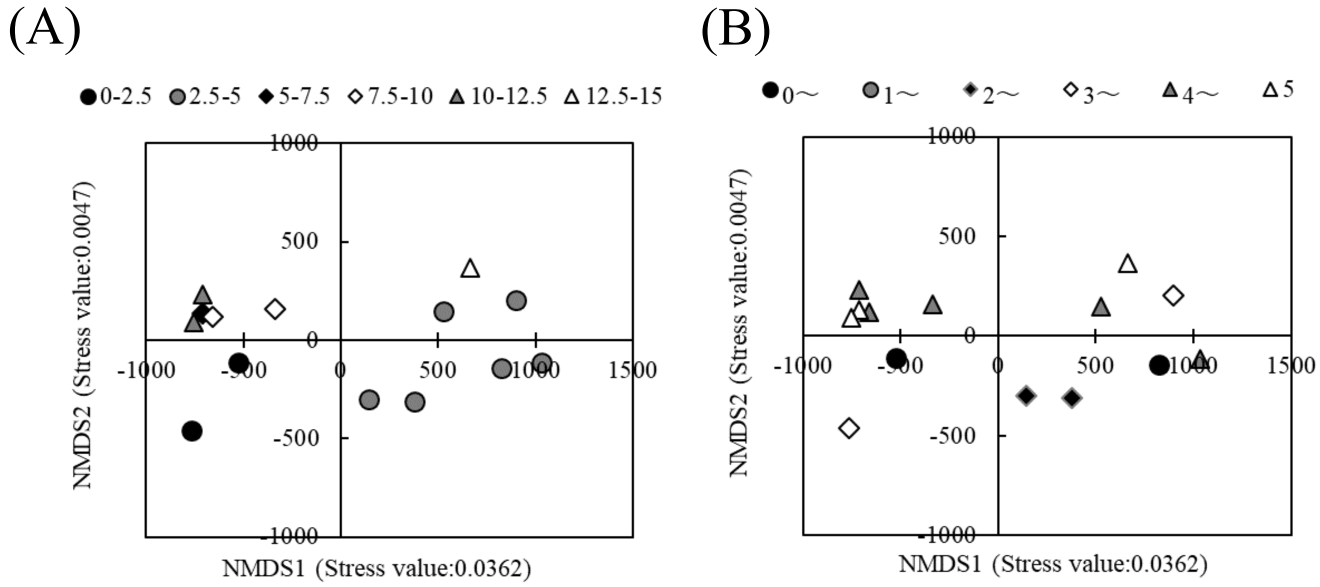

Fig. S2. Ordination of the selected 14 soil samples from croplands based on the chemical and biological characteristics by non-metric multi-dimensional scaling. pH, EC, contents of inorganic N, available P, exchangeable K, exchangeable Ca, exchangeable Mg, and humus, phosphate absorption coefficient, CEC, population densities of fungi, actinomycetes, bacteria, and *Fusarium* spp., and β-glucosidase activity were used for the analysis. The legend shows the median values of growth degrees of *Fusarium oxysporum* f. sp. *spinaciae* by the *Fusarium* co-cultivation method (mm) (A) and disease incidence of spinach by *F. oxysporum* f. sp. *spinaciae* (B) (n = 14).

Fig. S3

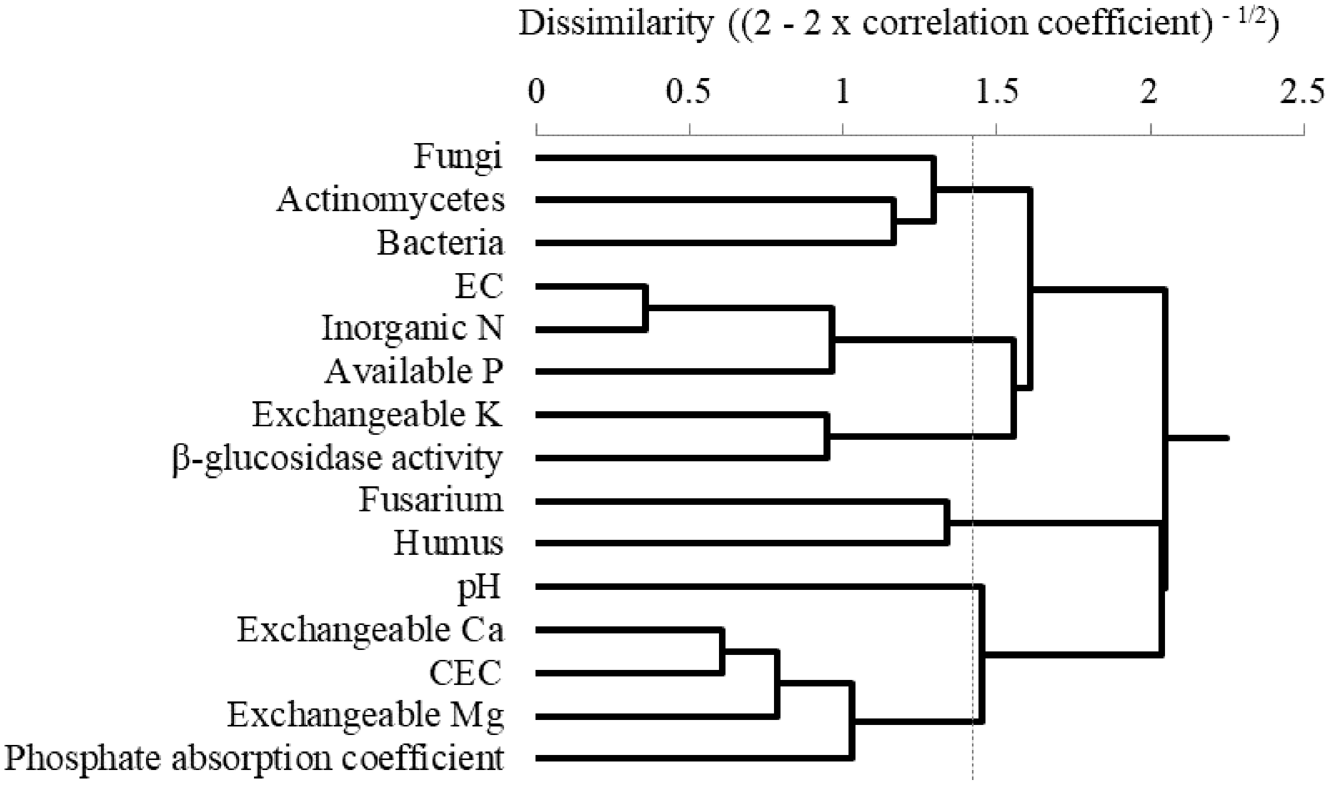

Fig. S3. Cluster analysis of the soil samples from croplands (n = 75) and experimental fields with long-term application of organic fertilizers (14) (n = 10) based on the chemical and biological characteristics for variable classification. Correlation coefficient between each variable was used as the distance between variables in the analysis. The dotted line indicates the dissimilarity value (1.45) used for the selection of the variables.

Fig. S4

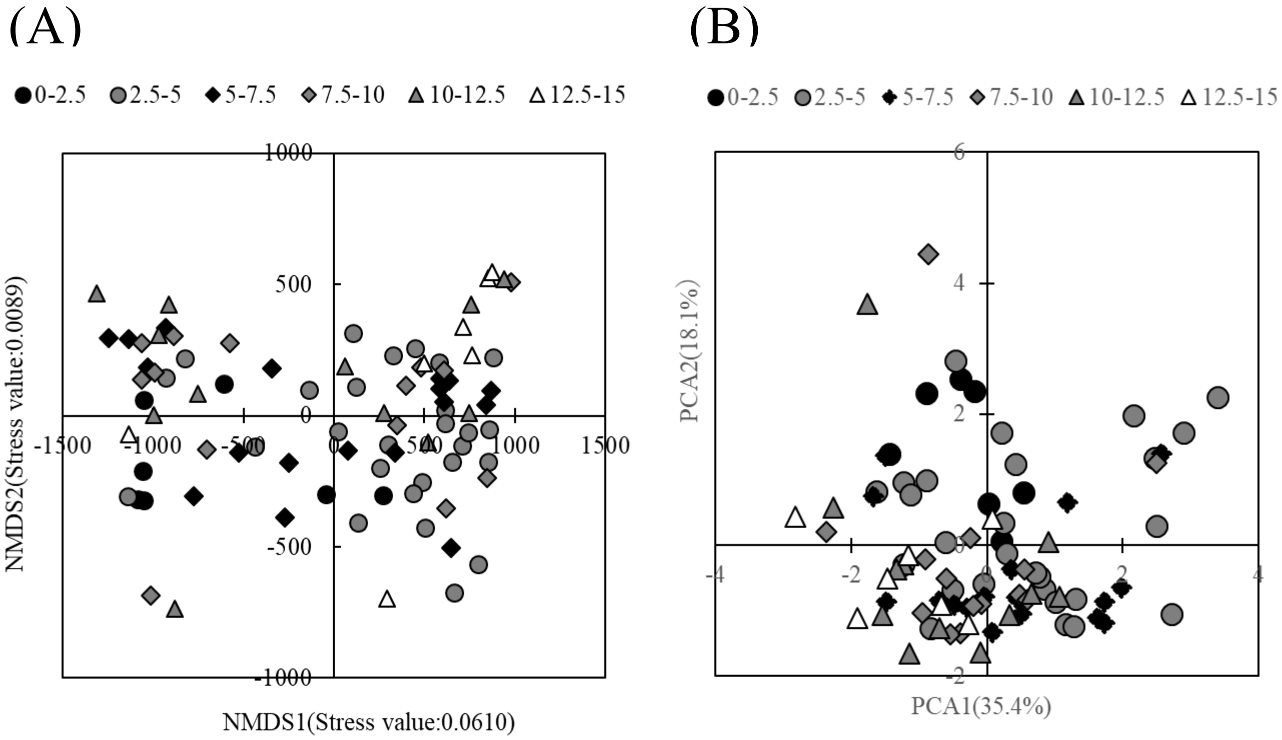

Fig. S4. Ordination of the soil samples from croplands (*n* = 75) and experimental fields with long-term application of organic fertilizers (15) (*n* = 10) based on chemical and biological characteristics using non-metric multi-dimensional scaling (A) and principal component analysis (B). Population densities of fungi, actinomycetes, bacteria, and *Fusarium* spp., pH, EC, contents of inorganic N, available P, exchangeable K, exchangeable Ca, exchangeable Mg, and humus, phosphate absorption coefficient, CEC, and β-glucosidase activity were used for the analysis (A). pH, EC, contents of exchangeable Ca and humus, population density of fungi, and β-glucosidase activity were used (B). The legend shows the median values of the growth degrees of *Fusarium oxysporum* f. sp. *spinaciae* by the *Fusarium* co-cultivation method (mm) (*n* = 85).

Table S1

Table S1. Chemical and microbiological characteristics of soils used in the experiment[a]

					Chemical characteristics															Microbial characteristics				
Place	Crop	Field	Sample number	Extension length of the colony (cm)	pH (H_2O)	EC (dS m^{-1})	Ammonia-N (mg kg^{-1})	Nitrate-N (mg kg^{-1})	Inorganic-N (mg kg^{-1})	Available P (P_2O_5 mg kg^{-1}) [b]	Exchangeable K (K_2O mg kg^{-1})	Exchangeable Ca (CaO mg kg^{-1})	Exchangeable Mg (MgO mg kg^{-1})	Phosphate absorption coefficient (P_2O_5 mg kg^{-1})	CEC ($cmol_c$ kg^{-1})	humus (%)	Total C (%)	Total N (%)	CN ratio	Bacteria ($\times 10^4$ cfu g^{-1})	Actinomycetes ($\times 10^4$ cfu g^{-1})	Fungi ($\times 10^3$ cfu g^{-1})	*Fusarium* spp. ($\times 10$ cfu g^{-1})	β-glucosidase activity (Amount of p-nitrophenol produced) (nmol hr^{-1} g^{-1} soil)
Yokote, Akita	Taro	Open ground (upland field)	1	3.75	6.48	0.12	8.3	10.3	18.6	900	833	4,965	838	13,008	19.3	3.9	1.71	0.18	9.6	2,800	1,900	120	300	371
			2	3.75	5.64	0.11	10.3	13.5	23.8	642	722	4,656	544	10,619	20.3	4.0	1.65	0.17	10.0	3,100	1,100	160	540	301
			3	11.50	7.29	0.16	9.3	43.4	52.7	692	775	6,468	630	13,018	19.9	3.9	1.68	0.17	10.0	960	480	41	34	331
Watari, Miyagi	Tomato	Green house	1	2.75	5.82	0.60	21.7	242.1	263.9	2,370	1,943	4,315	1,378	9,960	20.8	3.6	3.74	0.32	11.8	6,200	3,700	2,300	2	859
			2	3.00	5.88	0.50	39.2	184.5	223.7	2,010	842	4,206	1,135	10,515	16.7	3.0	2.57	0.24	10.9	3,600	3,300	2,100	2	747
Fukushima, Fukushima	Cucumber	Green house	1	2.75	6.43	0.24	23.7	68.9	92.6	3,446	634	4,228	994	11,779	17.7	3.7	2.57	0.25	10.2	4,700	3,200	170	710	578
			2	1.75	6.70	0.26	22.7	78.3	101.0	4,433	623	4,928	1,214	11,249	19.1	3.4	2.40	0.24	10.2	5,100	1,500	260	1,800	564
			3	4.50	7.89	0.23	11.3	11.3	22.6	1,214	817	5,554	1,098	13,383	17.0	2.6	1.48	0.15	10.2	4,400	1,600	100	25	807
			4	10.50	7.02	0.11	11.4	17.6	28.9	1,344	335	3,545	564	11,937	14.4	1.9	2.52	0.22	11.6	2,500	2,500	38	140	516
Kawachi, Tochigi	Cherry tomato	Green house	1	2.25	7.39	0.33	N.D.[c]	N.D.[c]	121.8	4,284	752	6,955	1,136	14,000	18.8	3.2	3.10	0.29	10.9	6,200	720	210	4	421
Ishioka, Ibaraki	Carrot	Open ground (upland field)	1	9.25	7.36	0.06	N.D.[c]	N.D.[c]	6.4	537	203	6,785	409	21,000	27.5	7.9	4.44	0.33	13.5	3,500	360	41	230	303
Omitama, Ibaraki	Carrot	Open ground (upland field)	1	5.00	6.47	0.06	N.D.[c]	N.D.[c]	10.2	305	690	3,253	590	18,000	19.5	6.7	2.54	0.22	11.4	1,090	840	49	270	368
Tsukuba Mirai, Ibaraki	Carrot	Open ground (upland field)	1	7.25	6.78	0.07	N.D.[c]	N.D.[c]	10.4	81	513	3,141	491	27,000	20.2	5.2	3.88	0.35	11.2	6,700	460	26	35	313
Tsuchiura, Ibaraki	Cucumber	Green house	1	4.00	5.53	0.56	14.0	178.3	192.3	3,341	469	4,953	755	18,814	28.4	5.4	3.17	0.31	10.2	820	3,100	55	2	420
			2	5.50	5.57	0.41	15.1	76.3	91.4	3,699	423	4,527	706	19,569	30.2	5.5	3.04	0.30	10.2	610	2,300	670	1	290
			3	9.25	6.03	1.11	27.6	412.5	440.1	4,324	3,862	9,533	2,006	24,053	40.8	4.6	5.25	0.58	9.0	1,700	2,500	10	2	613
			4	11.50	6.41	0.74	26.3	351.0	377.3	4,760	2,654	10,386	2,022	22,798	42.0	4.5	4.86	0.50	9.6	1,500	2,700	6	6	621
Tsuchiura, Ibaraki	Carrot	Open ground (upland field)	1	4.75	6.89	0.10	N.D.[c]	N.D.[c]	10.2	520	904	4,183	642	24,000	21.3	4.4	3.15	0.29	11.0	3,300	610	49	160	415
Namegata, Ibaraki	Carrot	Open ground (upland field)	1	3.50	6.87	0.05	N.D.[c]	N.D.[c]	9.9	99	599	3,318	516	23,000	20.2	4.7	2.49	0.24	10.5	2,600	660	180	760	416
Bando, Ibaraki	Lettuce	Open ground (upland field)	1	2.25	6.70	0.21	13.9	19.2	33.1	181	644	3,864	575	20,774	18.1	4.6	3.37	0.28	12.0	3,100	1,900	320	670	534
			2	1.00	6.63	0.34	9.8	50.0	59.8	98	526	4,683	663	24,748	19.3	4.1	3.27	0.28	11.7	4,000	4,000	470	740	599
Bando, Ibaraki	Green onion	Open ground (upland field)	1	10.00	6.32	0.20	13.0	40.0	53.0	108	898	3,728	684	24,330	22.7	6.3	3.82	0.31	12.2	2,500	2,000	200	230	713
			2	9.50	6.59	0.20	15.2	27.2	42.4	163	933	4,439	722	25,063	23.5	5.5	3.32	0.28	12.1	1,700	910	160	130	454
Yuki, Ibaraki	Cherry tomato	Green house	1	3.75	7.54	0.53	N.D.[c]	N.D.[c]	45.9	1,597	325	4,385	971	13,000	14.9	4.4	1.54	0.13	12.2	4,200	1,300	280	35	150
Yuki, Ibaraki	Carrot	Open ground (upland field)	1	13.25	7.83	0.11	N.D.[c]	N.D.[c]	7.9	351	794	6,662	735	25,000	24.8	4.6	3.34	0.32	10.5	2,400	990	61	110	275
Asahi, Chiba	Carrot	Open ground (upland field)	1	10.00	6.70	0.08	N.D.[c]	N.D.[c]	11.2	458	796	4,531	985	22,000	24.1	6.0	4.34	0.38	11.4	1,130	1,180	34	15	360
Yachimata, Chiba	Tomato	Green house	1	3.75	6.51	0.81	15.3	224.8	240.0	2,989	602	7,670	2,298	25,012	34.4	5.0	4.51	0.40	11.4	5,500	1,100	280	4	308
			2	1.25	6.62	0.66	10.9	165.1	176.0	2,744	438	7,663	2,302	24,472	32.0	4.9	4.39	0.39	11.3	5,100	1,300	260	22	220
			3	1.00	6.48	0.82	12.0	218.0	230.0	2,758	473	6,345	2,118	24,515	34.0	5.0	4.64	0.41	11.4	6,700	1,400	470	14	233
			4	2.00	6.50	0.75	13.1	263.1	276.2	3,100	679	7,423	2,294	24,357	33.6	4.8	4.26	0.39	10.9	4,800	410	460	26	236
Katori, Chiba	Spinach	Green house	1	6.25	7.24	0.11	N.D.[c]	N.D.[c]	14.3	2,099	1,411	7,207	1,724	22,000	33.9	6.8	5.54	0.51	10.8	2,300	730	42	20	563
Sanbu, Chiba	Cucumber	Green house	1	13.00	6.68	0.33	4.0	87.1	91.1	4,080	536	1,934	612	6,163	8.3	1.6	1.18	0.12	10.2	2,800	730	38	310	133
			2	12.75	6.41	0.46	6.1	120.5	126.6	4,252	500	1,924	635	5,948	8.4	1.6	1.19	0.13	9.4	2,400	750	38	150	118
			3	9.50	6.54	0.17	5.1	46.6	51.6	3,888	543	1,671	523	5,088	8.7	2.1	1.19	0.12	10.2	2,400	530	93	220	127
			4	10.75	6.83	0.16	7.1	24.3	31.4	3,652	331	1,780	567	5,387	8.2	1.9	1.15	0.11	10.5	1,600	540	59	160	105
Komatsu, Ishikawa	Rice	Open ground (paddy field)	1	6.50	5.85	0.04	12.4	2.1	14.5	155	263	1,886	294	14,207	16.9	9.3	3.77	0.29	13.0	850	420	310	220	644
			2	5.25	5.95	0.03	11.4	4.1	15.5	300	340	1,932	394	11,922	16.1	8.0	2.87	0.22	13.1	3,800	870	300	410	673
			3	10.25	6.11	0.03	8.2	5.1	13.4	247	303	2,326	461	7,707	14.7	6.3	2.35	0.18	13.4	1,200	630	61	200	549
			4	4.00	6.08	0.03	9.3	4.1	13.4	175	397	1,734	316	12,192	15.0	8.3	3.14	0.23	13.7	1,180	560	200	290	641
			5	4.75	5.55	0.03	9.2	1.0	10.2	123	274	1,032	284	7,202	7.4	1.3	0.96	0.10	9.8	580	260	64	210	624
			6	7.00	5.42	0.03	17.6	0.0	17.6	114	244	2,408	531	17,301	15.3	2.5	1.99	0.16	12.2	1,900	550	210	360	754
			7	13.75	6.98	0.05	10.2	1.0	11.2	368	391	2,514	778	9,798	11.8	4.4	1.40	0.11	12.9	1,600	490	13	2	333
			8	7.75	5.92	0.07	11.4	5.2	16.5	1,457	1,014	2,593	735	10,796	17.7	3.7	1.84	0.16	11.4	1,700	590	220	390	385
Takayama, Gifu	Pumpkin	Open ground (upland field)	1	9.00	6.40	0.07	8.2	3.1	11.2	838	551	2,809	325	9,755	12.6	5.0	1.79	0.15	12.0	2,300	440	210	680	315
			2	5.00	6.15	0.06	8.2	2.0	10.2	663	523	2,223	290	8,987	11.8	5.0	1.81	0.15	12.3	1,380	570	250	1,200	386
			3	7.00	6.52	0.07	8.2	4.1	12.2	765	795	2,641	443	8,490	12.3	4.7	1.70	0.14	11.9	1,900	410	73	560	395
			4	7.50	6.47	0.07	9.2	4.1	13.3	787	761	2,698	333	8,713	12.9	5.3	1.70	0.14	12.3	1,800	320	150	420	353
			5	6.00	6.57	0.08	9.2	3.1	12.3	553	363	2,787	296	8,901	13.0	5.1	1.75	0.14	12.3	1,250	530	190	360	423
Takayama, Gifu	Turnip	Open ground (upland field)	1	6.00	6.36	0.06	11.2	5.1	16.3	356	653	2,013	588	6,330	9.3	1.9	1.13	0.10	10.9	2,000	390	51	660	476
			2	8.00	5.87	0.06	13.4	8.3	21.7	227	1,191	2,626	748	11,681	15.4	3.8	1.80	0.15	11.7	710	440	59	550	556
			3	4.25	6.07	0.09	12.4	11.4	23.8	383	2,131	2,755	937	10,680	17.5	4.0	2.01	0.18	11.3	2,000	580	63	1,200	757
			4	8.75	6.17	0.12	14.3	16.4	30.7	707	1,389	2,888	979	6,657	15.8	4.4	3.70	0.33	11.4	3,600	560	48	470	759
			5	12.00	5.92	0.07	10.3	8.2	18.5	247	1,145	2,325	658	10,070	13.9	3.1	2.18	0.20	11.1	3,400	630	64	1,300	631
Gifu, Gifu	Broccoli	Open ground (upland field)	1	3.00	6.13	0.08	8.2	9.2	17.4	614	1,064	2,478	391	8,871	13.7	6.5	2.55	0.20	12.6	1,400	1,080	370	700	503
			2	2.50	6.12	0.08	11.2	4.1	15.3	583	877	2,157	359	7,744	11.7	5.4	2.48	0.20	12.3	940	2,200	370	710	616
			3	6.00	6.38	0.12	8.2	3.1	11.3	1,270	1,793	2,336	573	8,810	14.1	5.6	3.01	0.24	12.4	590	2,800	230	610	461
			4	4.75	6.48	0.11	8.2	5.1	13.3	818	1,076	2,250	528	8,145	12.9	4.8	2.64	0.21	12.4	6,400	2,100	260	1,600	661
			5	3.75	6.60	0.13	9.2	8.2	17.4	1,178	1,282	2,808	712	8,582	14.1	5.4	3.38	0.26	12.8	4,300	3,400	230	710	703
Tahara, Aichi	Chrysanthemum	Green house	1	6.75	6.47	0.28	11.5	67.9	79.4	3,791	638	7,749	1,764	16,383	27.5	3.7	2.32	0.28	8.3	3,600	470	44	69	325
			2	2.75	6.15	0.31	9.4	109.1	118.4	2,130	352	5,777	1,899	15,274	21.6	2.4	1.28	0.17	7.7	6,700	3,800	42	16	241
			3	2.50	6.23	0.38	18.6	115.9	134.5	3,517	618	5,659	1,119	13,461	21.5	3.6	2.53	0.28	9.1	51,700	2,300	34	31	380
			4	3.75	6.17	0.18	10.2	29.6	39.8	2,043	1,228	2,727	814	8,683	13.6	4.9	2.27	0.21	10.9	5,300	980	81	17	378
			5	3.25	5.96	0.95	7.1	329.4	336.5	3,325	1,303	3,040	1,208	9,532	11.7	3.8	1.95	0.23	8.5	5,100	1,400	50	14	378
Tahara, Aichi	Cabbage	Open ground (upland field)	1	6.00	5.42	0.14	11.2	15.2	26.4	487	546	1,371	295	6,879	8.6	2.6	0.99	0.10	9.6	2,500	770	260	1,900	528
Matsuzaka, Mie	Rice	Open ground (paddy field)	1	4.75	6.47	0.05	6.1	3.0	9.1	182	160	1,598	96	6,218	6.5	2.2	1.02	0.11	9.3	760	210	37	28	333
Suzuka, Mie	Fig	Open ground (upland field)	1	6.50	5.69	0.58	12.4	263.7	276.0	1,988	2,144	3,667	847	9,000	18.1	5.6	3.72	0.35	10.6	3,300	860	270	250	1,000
			2	2.75	4.75	0.49	15.5	255.7	271.2	938	1,456	2,299	566	8,880	16.9	5.8	3.75	0.34	11.0	4,700	1,180	190	280	549
			3	4.25	5.22	0.83	16.6	451.5	468.1	1,957	2,146	3,945	972	7,456	20.3	5.5	6.59	0.52	12.7	1,700	1,000	330	200	1,069
			4	2.75	5.95	0.57	12.4	273.5	286.0	2,134	1,683	5,222	792	8,599	20.5	5.4	5.00	0.47	10.6	2,000	1,900	170	290	1,130
			5	8.00	5.01	0.67	13.4	343.7	357.1	1,486	1,821	2,900	820	9,341	16.6	5.3	4.58	0.42	10.9	830	490	68	360	871
Kanoya, Kagshima[a]	Sweet potato	Open ground (upland field)	1	7.00	6.37	0.04	2.2	0.0	2.2	65	154	2,699	193	24,201	26.2	12.0	7.68	0.44	17.6	800	630	150	350	338
			2	11.50	6.11	0.06	2.2	0.0	2.2	54	58	1,850	45	24,129	24.5	12.0	7.46	0.42	17.8	1,100	480	53	240	285
			3	11.50	6.53	0.03	3.2	2.2	5.4	119	67	3,149	415	24,508	27.3	12.0	7.92	0.45	17.7	2,200	580	51	180	313
			4	9.25	6.41	0.04	3.3	2.2	5.5	109	264	2,723	464	23,759	26.7	12.0	7.57	0.46	16.5	940	590	52	220	358
			5	9.00	6.54	0.03	2.2	2.2	4.3	76	66	2,858	289	20,473	23.4	12.0	6.85	0.41	16.6	3,400	420	71	260	306

[a] pH, EC and microbial characteristics were measured in moist soil and the other characteristics were measured in air-dried soil.

[b] Truog method.

[c] N.D., not determined.
